# Editorial Comment: Impact of preoperative urodynamics on women undergoing pelvic organ prolapse surgery

**DOI:** 10.1590/S1677-5538.IBJU.2020.03.07

**Published:** 2020-02-20

**Authors:** D Glass, FC Lin, AA Khan, M Van Kuiken, A Drain, M Siev, B Peyronett, N Rosenblum, BM Brucker, VW Nitti, Cássio L. Z. Riccetto

**Affiliations:** 1 University of Chicago Department of Obstetrics and Gynecology ChicagoIL USA Department of Obstetrics and Gynecology, University of Chicago, Chicago, IL, USA; 2 University of California Los Angeles Division of Female Pelvic Medicine and Reconstructive Surgery Department of Urology Los AngelesCA USA Department of Urology, Division of Female Pelvic Medicine and Reconstructive Surgery, University of California Los Angeles, Los Angeles, CA, USA; 3 Mayo Clinic Department of Urology ScottsdaleUSA AZ Department of Urology, Mayo Clinic, Scottsdale, AZ, USA; 4 University of California Los Angeles Division of Female Pelvic Medicine and Reconstructive Surgery Department of Urology Los AngelesCA USA Department of Urology, Division of Female Pelvic Medicine and Reconstructive Surgery, University of California Los Angeles, Los Angeles, CA, USA; 5 New York University Division of Female Pelvic Medicine and Reconstructive Surgery Department of Urology New YorkNY USA Department of Urology, Division of Female Pelvic Medicine and Reconstructive Surgery, New York University, New York, NY, USA; 1 Faculdade de Ciências Médicas da Universidade Estadual de Campinas – UNICAMP Divisão de Urologia Feminina CampinasSP Brasil Divisão de Urologia Feminina - Faculdade de Ciências Médicas da Universidade Estadual de Campinas – UNICAMP, Campinas, SP, Brasil

## COMMENT

Pelvic organ prolapses (POP) are anatomical conditions of mainly surgical treatment. Thus, the role of urodynamics in POP preoperative assessment should be considered mainly as a tool for prediction of detrusor contractile function after POP surgical correction. Urodynamics also can be used as a complementary tool for the diagnose of occult urinary incontinence, although the same information can be reached through of a stress test with pessary or by reducing POP with a vaginal speculum. In fact the cost-effectiveness of urodynamics in preoperative POP workup still a matter of discussion (1).

In this retrospective study, the authors aimed to investigate the impact of urodynamic diagnosis in surgical planning of POP patients. For the analysis, preoperative indications for urodynamics were grouped in four categories: coexistence of urgency symptoms, mixed urinary incontinence, suspicion of occult urinary incontinence and coexistence of POP with symptoms of voiding dysfunction / incomplete emptying. They showed that among 316 women who underwent urodynamics prior to POP surgery, only 11 (3.2%) had a change in management and / or counseling not solely related to the presence or absence of occult stress incontinence. Furthermore, if patients with occult stress incontinence as an indication for urodynamics are excluded, management / counseling was carried out in only 9/146 (6.2%) of women. The authors concluded that excluding the mild utility in the diagnosis of occult stress incontinence, urodynamics had a limited role in POP management, despite highlight the usefulness of the urodynamic study in the pathophysiological diagnosis of voiding dysfunctions.
